# 

**Published:** 1880-06

**Authors:** 


BOOKS AND PAMPHLETS RECEIVED.

**Handbook of Physical Diagnosis.** By Dr. GUTTMANN, University of Berlin. Wm. Wood & Co.

**Examination of Urine.** By JAMES TYSON, M. D. 3d Edition. Presley Blakiston.

**Handbook of Medical Chemistry.** By WM. H. GREENE, M. D, University of Pennsylvania. H. C. Lee's Son & Co.

**Report of Public Schools of Maryland.**

**Anniversary Address before the N. Y. State Medical Society.** By H. D. DIDAMA, M. D.

**The Fallacies of Popular Clinical Medicine.** By JARVIS S. WRIGHT, M. D. Long Island College Hospital.

**The Black Arts in Medicine.** By JOHN D. JACKSON, M. M.

**The Utricular Glands of the Uterus** and the glandular organ of the new formation which is developed during Pregnancy in the Uterus of the Mammalia, including the Human Species. By Prof. GIOVANNI BATTISTA ERCOLANI, Permanent Secretary of the Academy of Sciences of Bologna, &c.; to which is appended his monograph upon the unity of the anatomical type of the Placenta in all the mammalia, and the physiological unity of the nutrition of the Fœtus in all the vertebrates. With a quarto atlas of fifteen plates, engraved by Bellini and reproduced by the Heleotype Process. Translated from the Italian under direction of Henry O. Marcy, A. M., M. D. Houghton, Osgood & Co., Boston. The Riverside Press, Cambridge. 1880.

